# Pattern of emergency department visits by elderly patients: study from a tertiary care hospital, Karachi

**DOI:** 10.1186/1471-2318-13-83

**Published:** 2013-08-12

**Authors:** Jabeen Fayyaz, Munawar Khursheed, Mohammed Umer Mir, UzmaRahim Khan

**Affiliations:** 1 Department of Emergency Medicine, Aga Khan University Hospital, Karachi, Pakistan

**Keywords:** Emergency department, Elderly visits, Pakistan

## Abstract

**Background:**

Worldwide the proportion of elderly people in the population is increasing. Currently in Pakistan 7.3 million people (5.6% of total population) are more than 60 years old. This age shift has emerged as an important health issue and is associated with an increased utilization of emergency services by the elderly. We carried out this study to assess the pattern of elderly patients (>60 years) who visit emergency departments in comparison to young adults (18–60 years).

**Methods:**

Data was collected retrospectively of patients aged 18 years or more who visited the Emergency Department (ED) of Aga Khan University Hospital, Karachi (AKUH) during September, 2009 to September, 2011. The data collection sheet included patient’s demographic information, triage category, reason for visit, clinical presentation, ED length of stay, day and time of presentation and their disposition. Data was entered and analyzed using SPSS version 19.0. Descriptive statistics were used to describe patient’s demographics. Chi-square (χ^2^) test was used as a test of significance to compare differences between groups for categorical data and t-test for continuous data. Multiple logistic regression analysis was done to find out the association between the patient characteristics and outcomes (admission and expiry).

**Results:**

Almost 24% (n = 13014) of all adults (n = 54588) presenting to the ED were over the age of 60 years. More than 57% of elderly patients belonged to the high priority triage category compared to 35% in younger patients. Most of the elderly patients ( 27%) presented with nonspecific complaints followed by shortness of breath (13%) and fever (9%). The median length of stay (LOS) in the ED for elderly was 379 minutes (252 min in under-60 yrs patients) and they were more likely to get admitted to in-patient departments compared to younger patients (OR 1.7 95% CI 1.6-1.8). A high proportion of those admitted (20%) required intensive or special care. Mortality in elderly patients was 2.3% as compared to 0.7% in young adults. This was accompanied by a higher mortality risk in the elderly with an odds ratio of 2.3 (CI 2–2.5).

**Conclusion:**

Elderly ED users differ significantly from younger adults in terms of criticality on presentation, ED LOS and final disposition.

## Background

A worldwide increase in the elderly population is an important concern as it has implications on the optimal delivery of health care especially in the emergency setting. The age shift contributes to issues such as Emergency Department (ED) overcrowding; older patients account for 12%-24% of all ED attendees worldwide [1]. Older patients tend to develop a more severe disease state and have co-morbidities leading to longer lengths of stay in the EDs, more resource utilization and higher admission rates to critical care areas [2,3]. In a study from USA, compared to younger patients, the elderly were 4.4 times more likely to use ambulance service, 5.6 times more likely to be admitted to a hospital, and 5.5 times more likely to be admitted to ICUs [4]. Center for disease control and prevention, in 2005 reported an increase of 16% of patients aged 50–64 years in the US [3].

In Pakistan, improved social conditions and advances in public health during the last half century have contributed to a significant increase in the average lifespan [5] Life expectancy at birth has improved to 62 years in men and 64 years in women [6,7] More people in Pakistan have promising mortality rates (over 60 years) than ever before [8,9]. Local data had shown that currently in Pakistan, 7.3 million people (5.6% of total population) are over 60 years old [10]. In 1951, this figure changed to approximately 1.92 million and it is projected to increase to 22.07 million by 2030; hence, increasing the dependency burden rate from 1.9 in 2000 to 19.8 in 2050 [11].

A survey carried out by Pakistan Medical and Research Council (PMRC) on the health of elderly population indicated them as frequent users of health care services [12]. A study assessing health needs of geriatric patients at the Aga Khan University Hospital, Karachi (AKUH) reported that 70% of elderly had five or more health problems, 75% had one or more chronic illnesses. Around 50% of them were on three or more medications [13].

### Rationale

International studies clearly demonstrate the special health care needs of elderly patients utilizing emergency services [14-20]. The present ED care models in Pakistan need to be modified to such an extent that we may be able to cope with the geriatric population who are tend to get sick more often along with multiple co morbidities [14]. Data on the patterns of emergency department visits; specifically in the elderly, are essential for developing an efficient health care delivery system. Our study delivers information on the basic characteristics of elderly patients presenting to local EDs for clinical management.

### Objective

To study the pattern of elderly patients (>60 years) who visited the emergency department in comparison to young adults (18–60 years) at tertiary care hospital of Pakistan.

## Methods

### Setting

**Figure 1:**
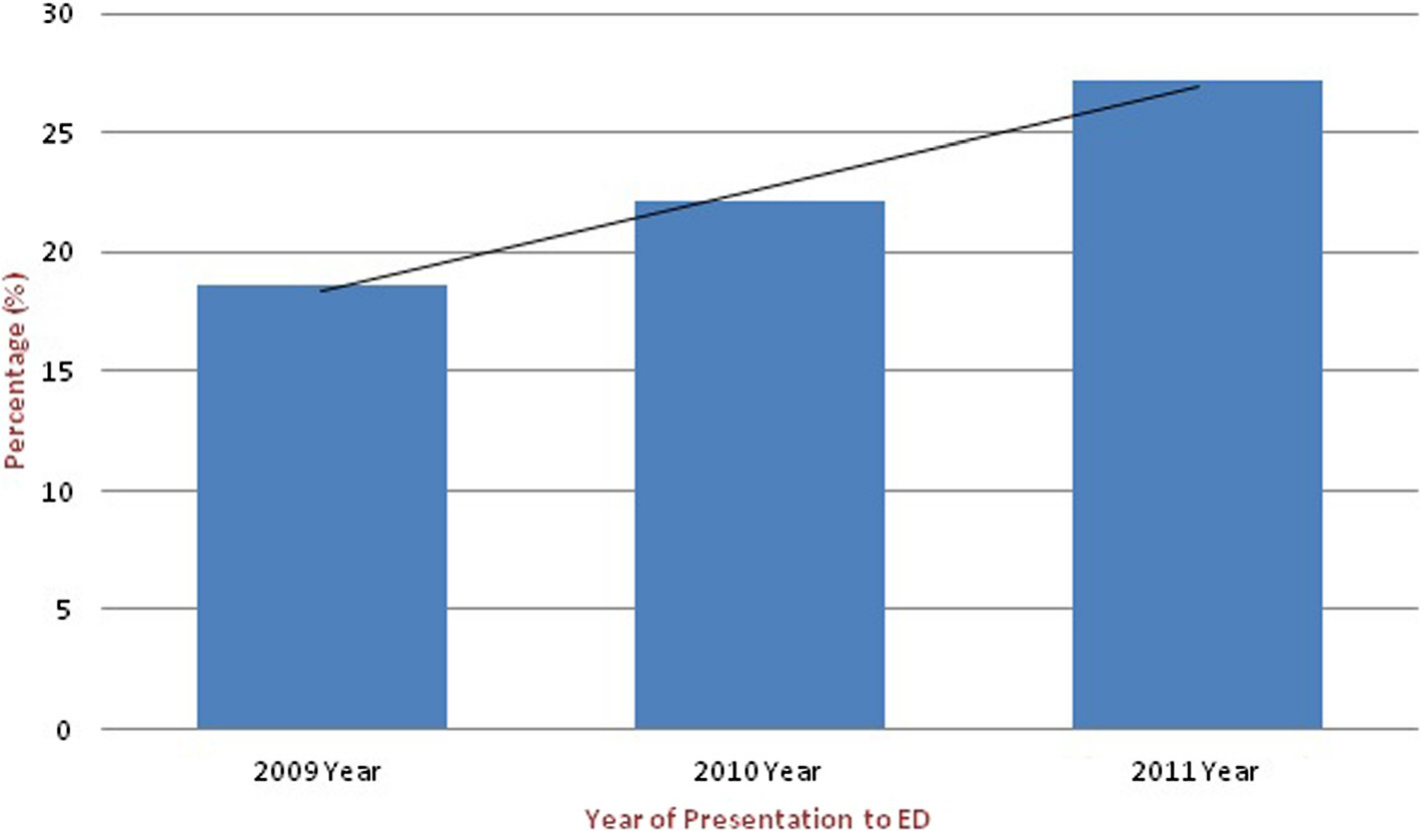
Trends of Elderly Visits in the ED from 2009–2011.

The study was conducted in the Emergency Medicine Department of the Aga Khan University Hospital (AKUH-ED) Karachi, Pakistan. AKUH is a 600 bedded, private tertiary care teaching hospital with an annual ED census of approximately 50,000 patients and a 37% admission rate. The ED has 46 patient care beds with pediatric, critical and non-critical care areas as well as an 8 bedded observation unit. It has a well-defined triage criteria using Emergency Severity Index-IV (ESI-IV) with an electronic patient’s data base system. (Figure 1) ESI IV is a five level triage system, P1 requiring immediate life -saving intervention, P2 high risk situation while P3-P5 according to the number of resource utilization.

All patients more above 18 years of age who were triaged in the AKUH-ED from September, 2009 to September, 2011 were included in the study. We used the electronic ED record system to extract data on demographic and clinical characteristics of patients. Information was collected on age, sex, triage priority level, presenting complaints, vitals, work-shift of the day week and month of visit, ED length of stay and final disposition ( admitted, discharge, LAMA or expired). Moreover, for patients who were admitted, we also collected data on level of care (ward, special care or Intensive care unit).

Patients were classified as dead on arrival (DOA) if they arrived in the ED in cardiac arrest and despite resuscitative attempts as per ACLS guidelines did not recover. Transferred out patients were those who after stabilization were transferred to another facility due to non-availability of high dependency beds in the hospital, financial constraints or on patients will. Patients were labeled as return visits if they had an un-scheduled visit in the ED within 48 hours of their initial visit. Data was available on 54,588 patients from 18 years onward which was then divided in two groups for comparison: young adults (18–60 years) and elderly (>60 years).

### Statistical analysis

Data of patients who were more than 18 years managed in ED was compiled and analyzed. The patients were divided into two groups: 18 to 60 years of age; and those older than 60 years old. Descriptive analysis was done and proportions were calculated for both groups. Chi-square tests for categorical variables (Triage category) and t-tests for the continuous variable (age) were applied to ascertain differences between the two groups. Multiple logistic regressions model was used to elucidate associations between patient characteristics and outcomes (admission to in-patient ward, and expiry). Two regression models were developed; one for admission to hospital and other for expiry. Initially, univariate analysis was carried out only for unadjusted odds ratios (ORs). All associations having a p-value of less than 0.25 were selected for the multivariate model. Multivariable analysis was used to calculate the adjusted ORs and the level of significance for the final models was kept at 0.05. Statistical Package for Social Sciences (SPSS) version 19 was used for the statistical analysis.

### Ethical approval

The study was reviewed and given approval by the Ethical Review Committee of the Aga Khan University, Karachi, Pakistan.

## Results

### General demographics

There were 86,122 visits to the AKU ED during the study period. Among them 41,574 (76.1%) belonged to the 18–60 years age group while 13,014 (23.8%) were over 60 years of age. Male to female ratio was 1.05:1 and 1.09:1 in both groups respectively with no statistically significant difference in the two groups with respect to gender. (Table 1) Our data also show an increasing trend in the number of elderly visits to the AKU-ED over time (Figure 1).

**Table 1:** Basic demographic characteristics of patients

	**18-60 yrs** **(N = 41,574)**	**> 60 yrs** **(N = 13,014)**	**p-value**
	**n (%)**	**n (%)**	
**Age (mean)**	37.2	70.9	< 0.001
**Gender**			
Female	20,202 (48.6)	6217 (47.8)	0.102
Male	21,372 (51.4)	6797 (52.2)	
**Triage Category**			
P1	5595 (13.4)	3032 (23.2)	< 0.001
P2	6045 (14.5)	3586 (27.5)	
P3	21,815 (52.4)	4603 (35.3)	
P4	6616 (16)	930 (7.1)	
P5	432 (1.1)	79 (0.6)	
**Day of presentation**			
Weekday	28,659 (68.9)	9162 (70.4)	0.002
Weekend	12,915 (31.1)	3852 (29.6)	
**Shift of presentation**			
7 am to 3 pm (Morning Shift)	13,282 (31.9)	4602 (35.4)	< 0.001
3 pm to 11 pm (Evening Shift)	16,655 (40.1)	5175 (39.8)	
11 pm to 7 am (Night Shift )	11,637 (28)	3237 (24.9)	
**Return visits**			
First visit	41,273 (99.3)	12,952 (99.5)	0.002
Bounce back visit	301 (0.7)	62 (0.5)	
**Disposition from ED**			
Admitted	9952 (23.9)	5131(39.4)	< 0.001
Dead on arrival	252 (0.6)	284 (2.2)	
Expired	297 (0.7)	300 (2.3)	
LAMA	2245 (5.4)	887 (6.8)	
Sent home	28,408 (68.3)	6235 (48)	
Transferred out	420 (1)	186 (1.4)	
**Inpatient care level**			
General	8837 (88.8)	4117 (80.2)	< 0.001
Intensive	378 (3.8)	324 (6.3)	
Special	737 (7.4)	690 (13.4)	
**Median length of stay in ED (in minutes)**			
P1	345	476	<0.001
P2	375	430	<0.001
P3	253	339	<0.001
P4	62	96	<0.001
P5	27	50	0.003

**Figure 2:**
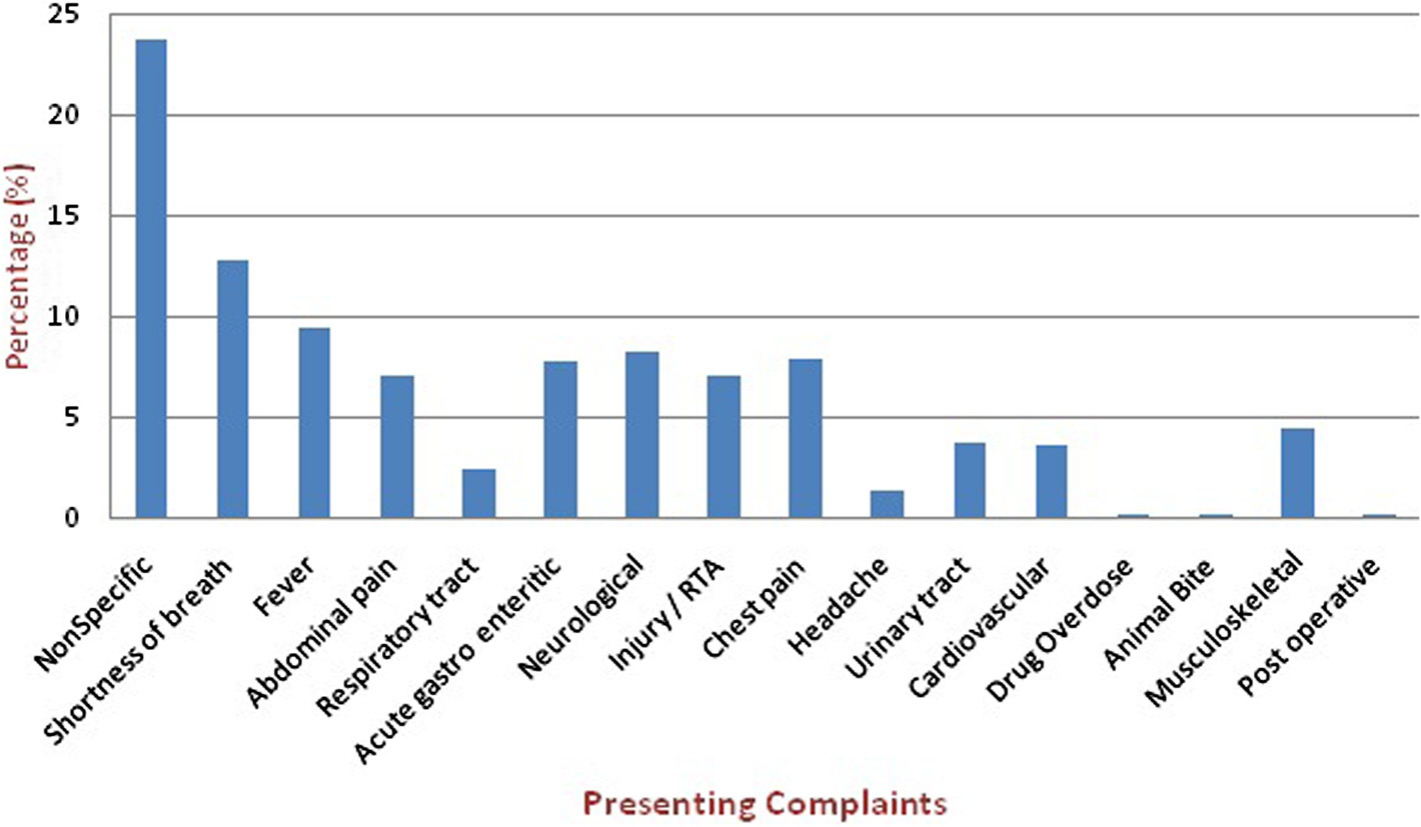
Presenting complaints of elderly patients.

Critical patients (P1 & P2) comprised a significantly higher proportion in the elderly (57%) compared to young adults (35%) (P-value < 0.001). This difference also reflected in the mortality proportions (2.3% vs. 0.7%). Elderly patients required admissions in 39.4% (5131). Almost 20% of the in-patient elderly required special or intensive care. Most patients visited during the evening clinical shift and return-visits were required in 0.5% (62) of elderly visits and 0.7% (301) in younger adults (p-value 0.002). Most of the elderly patients (27%) presented with nonspecific complaints followed by shortness of breath (13%) and fever (9%) (Figure 2).

#### Length of stay according to triage category

The median length of stay in the ED for elderly (379 minutes) was higher in all triage categories as compare to young adults (p-value < 0.001) (Table 1). The elderly had significantly higher proportions among the critical triage categories (P1 & P2) and had higher lengths of stay compared to the younger patients. The comparison of length of stay among the survived and expired elderly patients shows an inverse relationship between survival and length of stay in the ED (Table 2).

**Table 2:** Comparison of median Length of stay in ED (in minutes) among the expired and survived patients

**Triage Category**	**18 - 60 year olds**			**> 60 year olds**		
	**Expired**	**Survived**	**p-value**	**Expired**	**Survived**	**p-value**
**P1**	609	567	0.114	680	670	0.889
**P2**	692	588	0.047	575	618	0.41
**P3**	746	473	<0.001	808	579	0.097
**P4**	431	427	0.995	846	611	0.289
**P5**	-	836	-	-	499	-

We used multiple logistic regressions to ascertain which factors were associated with risk of being admitted to the hospital and dyeing. Two separate models were developed. After adjusting for other variables, the elderly were at higher risks of being admitted (OR1.7, 95% CI 1.6-1.8) and mortality (OR 2.3, 95% CI 2–2.5) as compared to the young patients. Other factors observed to have significant effects included gender, triage category and length of stay in the ED (Table 3).

**Table 3:** Regression models for admission and mortality of elderly (> 60 yrs) and young adults (18–60 yrs)

	**Unadjusted OR (95% CI)**	**p-value**	**Adjusted OR (95% CI)**	**p-value**
**Admission model**				
**Age**				
18- 60 yrs	(ref)		(ref)	
> 60 yrs	2.4 (2.3-2.5)	<0.001	1.7* (1.6-1.8)	<0.001
**Gender**				
Male	(ref)		(ref)	
Females	0.88 (0.85-0.91)	<0.001	0.95* (0.91-0.99)	0.018
**Triage category**				
P4 & P5	(ref)		(ref)	
P3	4.2 (3.8-4.6)	<0.001	4.1* (3.7-4.5)	<0.001
P2	11.8 (10.7-13.1)	<0.001	10.4* (9.4-11.5)	<0.001
P1	23.1 (20.8-25.6)	<0.001	20.6* (18.5-22.9)	<0.001
**Expiry model**				
**Age**				
18- 60 yrs	(ref)		(ref)	
> 60 yrs	3.7 (3.4-4.1)	<0.001	2.3^+^ (2.0-2.5)	<0.001
**Gender**				
Male	(ref)		(ref)	
Females	0.78 (0.71-0.87)	<0.001	0.89^+^ (0.79-0.99)	0.048
**Triage category**				
P4 & P5	(ref)		(ref)	
P3	1.5 (1.1-2.1)	0.019	1.2^+^ (0.85-1.7)	0.305
P2	6.2 (4.5-8.6)	<0.001	3.7^+^ (2.6-5.1)	<0.001
P1	20 (14.7-27.4)	<0.001	11.9^+^ (8.7-16.4)	<0.001
**Length of stay in ED**				
< 6 hrs	(ref)		(ref)	
>6 hrs	3.4 (3.1-3.9)	<0.001	1.8^+^ (1.6-2)	<0.001

* Also adjusted for Day of presentation and Shift of presentation.

† Also adjusted for Day of presentation, Shift of presentation.

## Discussion

Our study describes the patterns of elderly patient visits in comparison to the young adults visiting the ED of a tertiary care hospital in Pakistan. In low income countries due to limited primary healthcare systems, EDs serves as the front door for critical and noncritical illness for all, especially for the elderly population. Our study shows that 23.8% of all patients visiting the ED are more than 60 years old. These old age patients constitute only 5.3% of the general population [11]. A study from Taiwan shows similar results; 24.3% of all ED visits were of elderly patients while only 8.4% of the general population was elderly [21]. Such disproportionate usage by elderly is also supported by western studies that had shown increasing proportion of aging population. [4,15,21-23], although our study was based on a span of two years only, we still found an increasing trend in the elderly proportion of ED visits during this period.

Our study demonstrates that Elderly ED patients are of a higher acuity with 24.8% having life threatening conditions requiring resuscitative measures as compared to 13.8% in younger groups. This is comparable to the data from US showing 24.9% to 28.9% high acuity visits among the elderly in studies by Nawar, Wolinsky and others. [17,24,25] Elderly patients have higher rates of hospital admission and stay longer in the ED as compared to young adults which is comparable to a study from Taiwan where five in every ten elderly patients stay longer in the ED before final disposition. Reports from the US also indicate higher rates of admission (32%– 47%) in elderly compared to (7.5% -19%) in younger patients) [2,19,21,23]. The median length of stay (LOS) was significantly diverse among two groups with elderly patients staying more in ED. This may be because elderly patients are sicker and have multiple comorbidities, they require additional laboratory workup and often needed to be admitted but we are unable to address it in our study. When adjusted for the risk factors of being admitted, our study shows that overall high acuity patients were more likely to be admitted. As compared to young adults, elderly patients were 1.7 times more likely to be admitted. Hospitals were never thought as safe place for older population because data had shown ten times higher adverse outcome among hospitalized elderly patients as compared to young adults [26-29] while studying the Mortality model elderly patients had a higher risk of dying as compared to young adults. Overall patients with high acuity and those who are staying for more than 6 hours had an increased risk of dying. The international data had also supported the fact that the mortality in general is higher in those patients who stayed for longer duration before being shifted to ICU by 2.2 times [30]. Other temporal factors affecting mortality need to be studied further.

Interestingly 2.2% of elderly were dead on arrival in the ED and had no return of spontaneous circulation even after resuscitation according to Advance Cardiac Life Support (ACLS) protocol. This may either be due to prolonged illness; failure to recognize the signs of deterioration or because of lack of access to care .This needs to be investigated further as we could not look into this aspect due to limited data. Internationally, the outcome of Cardio Pulmonary Resuscitation (CPR) in elderly patients is reported to be as poor as only 3.8% to hospital discharge [31].

Return visits in elderly patients in this study were comparatively less than the international reported rates [32-34]. A possible explanation could be that physicians have a tendency to admit elderly patients as most of them have non-specific complaints [35,36].

### Limitation

Our study relied on data from the electronic information database of AKU. Complete clinical records were not available in the system. Information such as co-morbidities, final diagnosis and resource utilization (e.g., lab and radiological investigations, consultations, medications etc.) for these patients could not be ascertained in the current study design. Therefore, potential correlations of these factors with length of stay and mortality were not explored. The age range above 60 years is broad and heterogeneous in health and functional profile; there is a strong need of studies that focus at the pattern of visits and their outcomes by stratification. We assumed uniform quality of clinical care for all patients coming to the AKU-ED. The quality of care can have an effect on patient outcomes but could not be adjusted in our regression analysis due to limited availability of information.

## Conclusion

The Elderly population has significantly different health care needs than young adults which require special attention. They are sicker, stay longer, and require more admissions in high dependency unit as compared to their younger fellows. An increasing trend of ED utilization by elderly has obvious implications for designing a system of dedicated geriatric care in ED with specific triage process addressing their special need. Further focused studies addressing the relationship of triage category with LOS and its impact on short and long term outcome are required.

## Abbreviations

ED: Emergency department; OR: Odd ratio; CI: Confidence interval; AKUH: Aga Khan University Hospital; P1: Priority level 1; P2: Priority level 2; P3: Priority level 3; P4: Priority level 4; P5: Priority level 5; ACLS: Advance cardiac life support; ICU: Intensive care unit; PMRC: Pakistan medical & research council; LAMA: Leave against medical advice; ERMS: Emergency room management system; CPR: Cardio pulmonary resuscitation; ROSC: Return of spontaneous circulation; SPSS: Statistical package for social sciences.

## Competing interests

The authors declare that they have no competing interests.

## Authors’ contributions

JF and MK contributed equally to the work. MUM and URK participated in the design, and data analysis. JF, MK and MUM drafted the manuscript. URK also done the final reading .All authors read and approve the final manuscript.

## Pre-publication history

The pre-publication history for this paper can be accessed here:

http://www.biomedcentral.com/1471-2318/13/83/prepub
